# The effect of resistance training set configuration on strength and muscular performance adaptations in male powerlifters

**DOI:** 10.1038/s41598-021-87372-y

**Published:** 2021-04-12

**Authors:** Hamid Arazi, Amin Khoshnoud, Abbas Asadi, James J. Tufano

**Affiliations:** 1 Department of Exercise Physiology, Faculty of Sport Sciences, University of Guilan, P.O. Box 41635-1438, Rasht, Iran; 2 Department of Physical Education and Sport Sciences, Payame Noor University, Tehran, Iran; 3 Faculty of Physical Education and Sport, Charles University, Prague, Czechia

**Keywords:** Environmental sciences, Orthopaedics, Physiology

## Abstract

The purpose of this study was to determine the effects of different set configurations on strength and muscular performance adaptations after an 8-week resistance training program. Twenty-four male powerlifters participated in this study and were randomly assigned to one of two resistance training groups: (1) cluster sets (CS: n = 8), (2), traditional sets (TS: n = 8), and a control group (CG: n = 8). All powerlifters were evaluated for thigh and arm circumference, upper and lower body impulsive activities, and 1 repetition maximum (1RM) in the back squat, bench press, and deadlift prior to and after the 8-week training intervention. After training, both the CS and TS groups increased arm and thigh circumferences and decreased body fat. The CS group resulted in greater increases in upper and lower body impulsive activities than the TS group, respectively. In addition, the CS and TS groups indicated similar changes in 1RM bench press, back squat, and deadlift following the 8 weeks training intervention. These results suggest that cluster sets induce adaptive changes that favor impulsive activities in powerlifters.

## Introduction

It has been well documented that resistance training (RT) is an optimum training modality for improving muscular strength and impulse-dependent performance^1^. Considering the importance of strength for powerlifters during daily training and competition, designing RT programs for powerlifters requires an optimal combination of several variables including exercise selection, exercise order, training frequency, training load, number of repetitions, and rest interval between sets to produce greater increases in maximal strength for the three competition lifts (i.e., bench press, back squat and deadlift)^2^. Regarding the latter acute program variable, some studies have focused on the effects of altering the set structure on performance adaptations by adjusting rest intervals between sets, within sets, or both^3–6^. Specifically, it has been proposed that the use of a cluster set configuration (i.e. adding extra rest intra-set rest periods) can facilitate restoration of the metabolic and excitatory cellular environment during RT sessions for better management of fatigue during training sessions^6–9^.

To date, there are numerous studies which have explored the acute effects of cluster sets on various mechanical factors, but there is limited research examining the longitudinal effect of cluster sets on physiological and performance adaptations. In one of the first longitudinal studies in the field of cluster-set resistance training, Oliver et al.^10^ reported that redistributing a portion of each inter-set rest period to the middle of each set resulted in greater strength and hypertrophic gains when compared to traditional sets following a 12-week RT program. Asadi and Ramirez-Campillo^4^ later examined the impact of using cluster sets during a 6-week plyometric training program and found large improvements in countermovement jump performance, while the traditional set training program demonstrated only moderate improvements. Morales-Artacho et al.^11^ examined the role of set structure as part of a 3-week resistance training program and determined that a using cluster sets was more efficient for inducing velocity and jump adaptations specific to the loads used during training. Recently, Arazi et al.^3^ examined the effects of using cluster sets as part of an 8-week RT program on strength and jumping performance in female volleyball players and reported greater vertical jump performance in the group that trained with cluster sets, whilst there were no differences in the strength gains between the athletes who trained with traditional or cluster sets as part of their RT program.

Collectively, these studies suggest that resistance training programs that use traditional sets are considered more effective^10,12^ or equally effective^4^ at maximizing maximal strength gains, while programs that contain cluster sets are more effective at maximize rate of force development^3,4,11^. However, other research suggests that limiting acute neuromuscular fatigue, similar to what occurs with cluster sets^13^, can induce similar or greater strength and jumping adaptations as more fatiguing protocols^14,15^ that are more indicative of traditional sets. It is understood that impulse generated by athletes is important for jumping and sprinting performance^10^. In addition, there is a significant relationship between strength levels and rate of force development, indicating that skeletal muscle mechanics that form the force–velocity relationship dictate that maximal strength plays a role in muscular impulse momentum. Thus, the improving or optimizing impulse is influenced by a multitude of factors in addition to maximal strength^15^. Based upon these confounding findings regarding acute neuromuscular fatigue and strength adaptations, combined with the limited longitudinal research examining cluster sets in athletes, further research should be conducted to investigate the effect of set structure on strength adaptations to expand the knowledge available in this area.

Specifically, there is a lack of research on the effect of cluster set structures on specific groups or populations who have unique performance requirements. For example, the competitive sport of powerlifting requires maximal muscle strength (i.e., one repetition of maximum) in the back squat, bench press, and deadlift exercises. Therefore, performing RT using an optimum set configuration could be important to increase muscle strength. Although cluster sets are often performed to improve jumping ability^7,8^, some research also indicates that they may be equally as effective^3^ or even superior to traditional sets^10^ for developing strength as well. In addition, as there are positive correlations between strength and rate of force development and impulse generated by athletes, it seems that the development of impulse-momentum following the training (i.e., cluster sets) could be an added benefit of training that could also improve the performance of many athletic movements that influenced by a multitude of factors^14^. Despite individuals who can produce high forces in a short amount of time often displaying great strength level, little is known about the effects of RT with set configuration (i.e., power-type RT) on strength and impulsive activities in powerlifters.

In fact, to the authors’ knowledge there are no studies that have examined the longitudinal (i.e., 8 weeks training) impact of using cluster sets on strength and impulsive activities in powerlifters. Considering the conflicting results of previous studies and although many coaches believe that cluster sets should not be used as part of a powerlifting programs because the sport primarily relies on the ability to express high levels of maximal strength, no studies directly confirm this notion. Nevertheless, it appears that successful performance of many athletic movements is influenced by various factors and the development of impulse-momentum could enhance strength performance of athletes^16^. Therefore, the purpose of this study was to determine the effects of an 8-week RT program with differing set configurations (i.e. cluster and traditional sets) on anthropometrics, strength, and impulsive activities in college-aged male powerlifters. Based on previous studies, it was hypothesized that RT with traditional sets would result in more favorable strength development compared to cluster sets, but cluster set training would result in greater impulsive activities adaptations than traditional sets in powerlifters.

## Results

**Figure 1:**
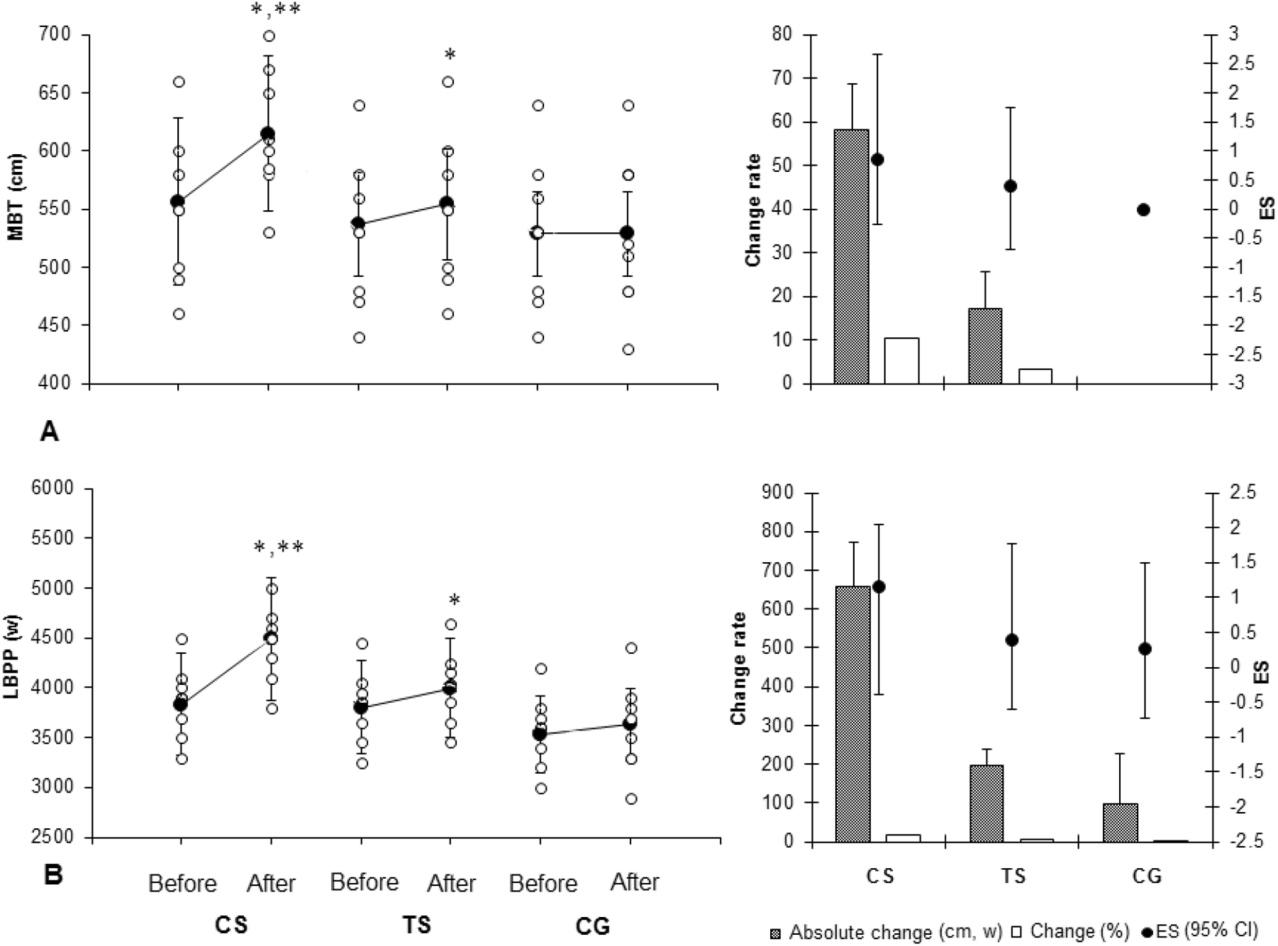
Changes in impulsive activities in response to 8 weeks training intervention (mean ± SD). CS: cluster sets, TS: traditional sets, CG: control group. MBD: medicine ball throw, LBPP: lower body peak power.*Denotes significant within-group differences between before and after-training values (*p* ≤ 0.05). **Denotes significant differences between the CS and TS groups after-training (*p* ≤ 0.05). (Drawn using Microsoft Excel software, version 2010; https://www.microsoft.com/en-us/microsoft-365/previous-versions/microsoft-excel-2010).

**Figure 2:**
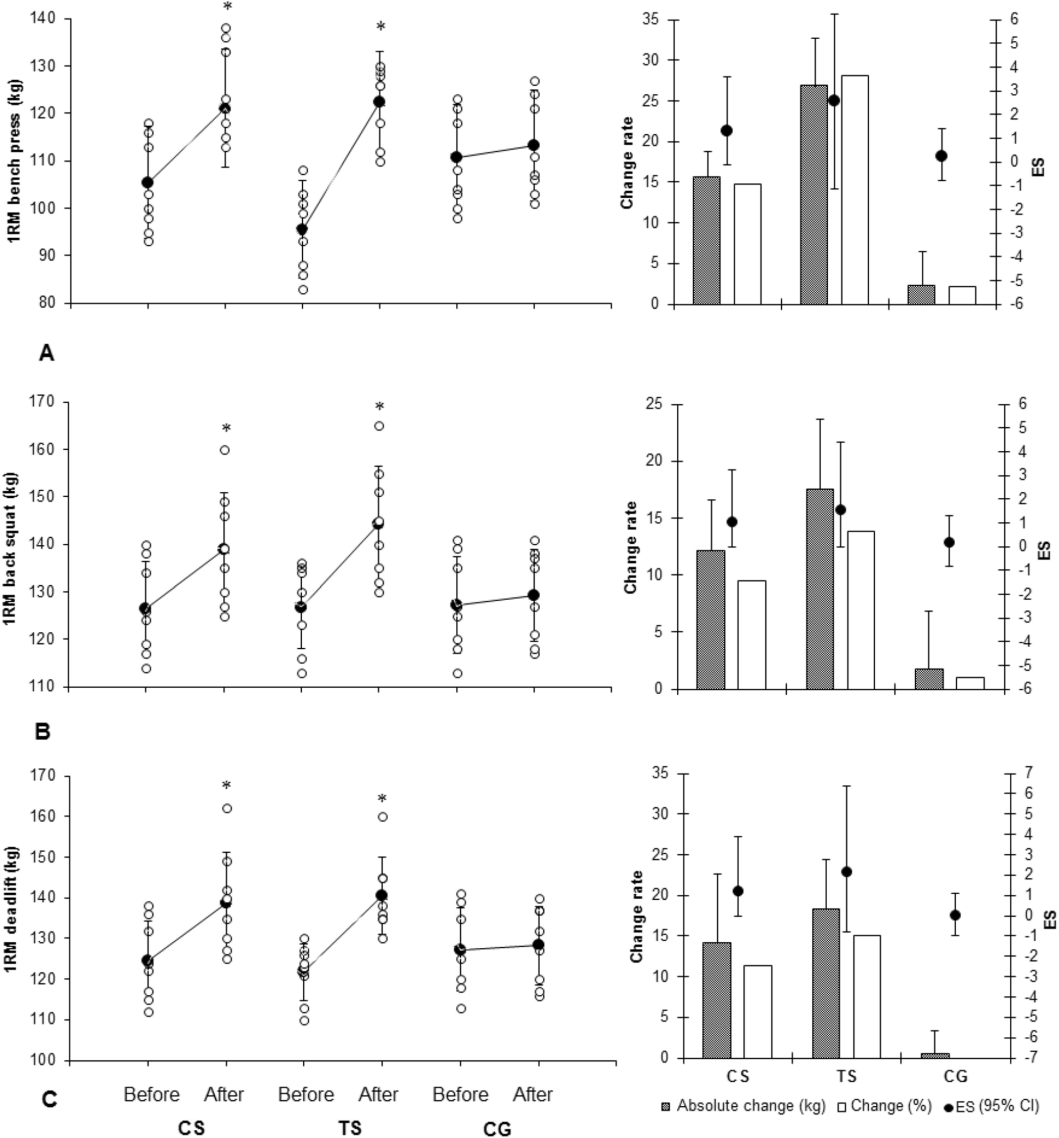
Changes in strength performance variables in response to 8 weeks training intervention (mean ± SD). CS: cluster sets, TS: traditional sets, CG: control group. 1RM: one repetition maximum.*denotes significant within-group differences between before and after-training values (p ≤ 0.05). **denotes significant differences between the CS and TS groups after-training (*p* ≤ 0.05). (Drawn using Microsoft Excel software, version 2010; https://www.microsoft.com/en-us/microsoft-365/previous-versions/microsoft-excel-2010).

Before training, no significant differences were observed between groups in dependent variables (*p* > 0.05). No significant changes in the CG were observed in any variable after training period (*p* > 0.05) (Tables 1, 2 and Figs. 1, 2).

**Table 1:** Absolute and relative 1RMs for each lift.

Characteristic		CS (n = 8)	TS (n = 8)	CG (n = 8)
		Mean ± SD	Mean ± SD	Mean ± SD
1RM BP (kg)	Before	105.4 ± 11.8	95.4 ± 10.3	110.7 ± 11.2
	After	121.0 ± 12.4*	122.3 ± 10.7*	113.1 ± 11.7
1RM BP/BW (Ratio)	Before	1.34 ± 0.81	1.22 ± 0.52	1.31 ± 0.63
	After	1.46 ± 0.67*	1.49 ± 0.73*	1.34 ± 0.78
1RM BS (kg)	Before	126.5 ± 9.8	126.7 ± 8.7	129.5 ± 13.7
	After	138.8 ± 12.1*	144.1 ± 12.2*	130.7 ± 14.0
1RM BS/BW (Ratio)	Before	1.56 ± 0.87	1.55 ± 0.84	1.55 ± 0.83
	After	1.72 ± 0.79*	1.78 ± 0.64*	1.58 ± 0.71
1RM DL (kg)	Before	124.5 ± 9.8	121.7 ± 6.9	127.5 ± 11.7
	After	138.7 ± 12.4*	140.5 ± 9.4*	128.1 ± 10.7
1RM DL/BW (Ratio)	Before	1.51 ± 0.54	1.47 ± 0.43	1.55 ± 0.73
	After	1.69 ± 0.73*	1.74 ± 0.65*	1.59 ± 0.69

*CS* cluster sets, *TS* traditional sets, *CG* control group, *1RM* one repetition maximum, *BP* bench press, *BS* back squat, *DL* deadlift, *BW* body weight. Values are mean ± SD. *Denotes significant within-group differences between before and after-training values (*p* ≤ 0.05).

**Table 2:** Changes in anthropometric variables in response to 8 weeks training intervention (mean ± SD).

Variables	CS(n = 8)		TS (n = 8)		CG(n = 8)	
**Weight (kg)**						
Before	78.6 ± 4.5		79.2 ± 4.1		77.2 ± 4.9	
After	79.9 ± 4.2		80.5 ± 4.7		78.2 ± 4.9	
ES	0.3 (− 0.7 to 1.27)	Small	0.29 (− 0.71 to 1.26)	Small	0.2 (− 0.79 to 1.18)	Small
Absolute change	1.6 ± 2.5		1.3 ± 2.3		1.2 ± 2.9	
% change	1.6		1.6		1.2	
**Body fat (%)**						
Before	17.9 ± 3.1		18.3 ± 3.1		16.7 ± 2.3	
After	16.9 ± 3.1	*	17.4 ± 2.9	*	17.2 ± 2.6	
ES	− 0.32 (− 1.29 to 0.68)	Small	− 0.31 (− 1.27 to 0.70)	Small	0.2 (− 0.79 to 1.17)	Small
Absolute change	− 1.0 ± 0.3		− 0.9 ± 0.5		0.5 ± 0.5	
% change	− 6		− 5		3.1	
**Thigh circumference (cm)**						
Before	57.5 ± 3.1		57.1 ± 2.7		56.0 ± 3.0	
After	59.5 ± 4.1	*	59.5 ± 2.5	*	55.1 ± 3.5	
ES	0.55 (− 0.48 to 1.52)	Small	0.92 (− 0.15 to 1.90)	Moderate	− 0.28 (− 1.25 to 0.72)	Small
Absolute change	2 ± 2.5		2.3 ± 1.3		− 0.9 ± 0.7	
% change	3.1		4.2		− 2.1	
**Arm circumference (cm)**						
Before	35.4 ± 2.0		36.5 ± 1.5		36.8 ± 1.1	
After	36.8 ± 2.1	*	37.9 ± 2.2	*	36.7 ± 1.1	
ES	0.68 (− 0.36 to 1.65)	Moderate	0.74 (− 0.31 to 1.71)	Moderate	0.09 (− 0.89 to 1.07)	Trivial
Absolute change	1.4 ± 0.5		1.3 ± 0.6		− 0.1 ± 0.5	
% change	4.1		4.2		0	

*CS* cluster sets, *TS* traditional sets, *CG* control group. *Denotes significant within-group differences between before and after-training values (*p* ≤ 0.05). *G* group, *T* time. a, b, c denotes trivial, small, and moderate effect size (ES), respectively.

### Anthropometric measures

After the 8-week training intervention, neither the CS nor TS group displayed significant changes in body weight (*p* = 0.720). Whereas, for percent body fat, both the CS (ES =  − 0.32 95% CI =  − 1.29 to 0.68) and TS (ES =  − 0.31 95% CI =  − 1.27 to 0.70) groups showed significantly (*p* = 0.042) small decreases after 8 weeks training. There were no statistically significant group × time (*p* = 0.17) interactions in body fat percentage (Table 2). Significant main effects of time (*p* = 0.041), but not group × time (*p* = 0.14) interactions were observed for the arm and thigh circumferences, respectively. The CS group demonstrated significant small increases in the thigh (ES = 0.55, 95% CI =  − 0.48 to 1.52) circumference and moderate increases in the arm (ES = 0.68, 95% CI =  − 0.36 to 1.65) circumference. In addition, the TS group demonstrated significant moderate increases in the thigh (ES = 0.92, 95% CI =  − 0.15 to 1.90) and arm (ES = 0.74, 95% CI = -0.31 to 1.71) circumferences (Table 2).

### Impulsive activities measures

There was a significant main effect of time (*p* = 0.001) and a group × time (*p* = 0.001) interaction for the MBT and LBPP performance which revealed that at the post-testing period the CS group increased their ability to generate high forces in a short period of time to a greater extent than the TS group (*p* = 0.021). The CS group demonstrated significant moderate increases in the MBT (ES = 0.84, 95% CI =  − 0.23 to 1.81) and LBPP (ES = 1.15, 95% CI =  − 0.04 to 2.14) performance, but the TS group demonstrated significant small increases in the MBT (ES = 0.39, 95% CI =  − 0.62 to 1.36) and LBPP (ES = 0.40, 95% CI =  − 0.61 to 1.37) performance (Fig. 1A and B).

### Strength measures

There was a significant main effect of time (*p* = 0.010), but not a group × time (*p* = 0.740) interaction for 1RM bench press. In addition, the CS group showed large training effects (ES = 1.29, 95% CI =  − 0.15 to 2.29), while the TS showed very large training effects (ES = 2.56, 95% CI = 1.13 to 3.71) in 1RM bench press following 8 weeks training (Fig. 2A).

There was a significant main effect of time (*p* = 0.020), but not group × time (*p* = 0.311) interactions for the 1RM back squat (Fig. 2B). In addition, the CS group showed moderate training effects (ES = 1.06, 95% CI = 0.01 to 2.1), while the TS showed large training effects (ES = 1.55, 95% CI = 0.43 to 2.67) in 1RM back squat following 8 weeks training (Fig. 2B).

There was a significant main effect of time (*p* = 0.011), but not group × time (*p* = 0.656) interactions for the 1RM deadlift (Fig. 2C). In addition, the CS group showed moderate training effects (ES = 1.2, 95% CI = 0.14 to 2.27), while the TS showed very large training effects (ES = 2.16, 95% CI = 0.92 to 3.39) in 1RM back squat following 8 weeks training (Fig. 2B). In addition, the relative strength increased for both the CS (*p* = 0.001) and TS (*p* = 0.001) groups following the training period, but post hoc analyses indicated no significant differences between the TS group and CS group (*p* > 0.05) (Table 1).

## Discussion

The aim of the present study was to compare the effects of cluster sets and traditional sets during an 8-week off-season powerlifting program on measures of muscular adaptations (i.e., thigh and arm circumferences, percent body fat, upper and lower body impulsive activities, and bench press, back squat, and deadlift 1RM. Our study suggests that both training interventions were effective to improve muscle size, impulsive activities, and muscular strength; however, the CS group demonstrated significantly greater improvements in both impulsive activities tests (i.e., MBT and LBPP) than TS group. In contrast, the TS group demonstrated significantly greater improvements in lower body muscular strength (i.e., squat, and deadlift 1RM) than CS group, which are in line with our hypothesis. In addition, relative strength increased following the training period and the TS group indicated significantly greater adaptive changes than CS group in the 1RM BS/BW ratio.

It is generally supported that using traditional sets during RT is suitable for improving muscular strength^7^. In this study, we found that both the CS and the TS groups increased strength after 8 weeks training. Although, the results indicated no significant differences between the CS and TS groups in strength measures, the TS group showed more training effects in 1RM bench press (ES = 2.56 vs. 1.29), 1RM back squat (ES = 1.55 vs. 1.06) and 1RM deadlift (ES = 2.16 vs. 1.2) compared with the CS group. Regarding the magnitude of training effects, greater improvements in muscular strength in response to traditional sets were supported. Rooney et al.^12^ and Lawton et al.^16^ reported significantly greater increases in bench press strength with traditional sets when compared to cluster sets, but Oliver et al.^10^ reported that the use of cluster sets could result in greater improvements in muscular strength in response to RT performed for up to 8 to 12 weeks via greater neuromuscular activation following cluster sets training at 65 to 75% 1RM^10^. The reason for the discrepancy with Oliver et al.^10^ findings' could be due to training duration (8 vs. 12 weeks, greater adaptive changes following a longer training duration), training intensity (70 to 90% 1RM vs. 65 to 75% 1RM, greater motor unit recruitment with heavier load), and fitness level of subjects (healthy subjects vs. powerlifters, greater adaptive responses in strength to RT in previously trained subjects). Improvements in muscular strength following the RT could be due to increases in various neuromuscular adaptations^1,17^; however, the increases in body weight and muscle size in this study could explain another possible reasons to increase strength gains following the 8 weeks training.

In agreement with previous studies^9,18^ and in accordance with our original hypothesis, CS resulted in greater upper and lower impulsive activities compared to TS (MBT, ES: 0.84 vs. 0.39, % change: 10.5% vs. 3.2%, and LBPP, ES: 1.15 vs. 0.4, % change: 17.1% vs. 5.1%). In line with our findings, other studies have also reported that training using cluster sets is more effective than traditional sets for improving jump performance^3,4,18^. Recently, Morales-Artacho et al.^11^ and Arazi et al.^3^ examined the effect of 3 and 8 weeks cluster sets and traditional sets RT on impulsive activities and found greater adaptive responses in vertical jump performance following cluster set training. The possible explanation of the greater adaptive responses in impulsive activities using cluster sets may be greater velocities in RT due to lower accumulation of fatigue over the course of a set, and the reduced metabolic responses may allow one to move the barbell faster^3,4,7–9^.

Lastly, the present study demonstrated small decreases in body fat percentage for both the CS and TS following 8 weeks of training. Additionally, the TS group indicated moderate increases in arm and thigh circumferences after training, but the CS group showed only a small increase in the arm and moderate increase in the thigh circumferences. Therefore, increases in body weight and muscle size with reducing in body fat could be reflective of increases in myofilaments, actin and myosin filaments, sarcoplasm, and connective tissue^19,20^. In addition, the results of this study are in line with previous reviews and meta-analyses that addressed the beneficial effects of RT on muscle hypertrophy^1,2,17,20^, which are in accordance with our hypothesis.

In conclusion, as results from the study 8 weeks of RT increased muscular strength and impulsive activities in powerlifting athletes and it may be useful to incorporate cluster sets during designing RT program to improve jump performance, but the data of the present study confirms that that strength and conditioning professionals in the field of powerlifting use traditional sets to improve strength performance; however, the results of strength measured did not show statistically significant differences between the TS and CS group, the magnitude of training effects (i.e., ES) were greater for the TS than the CS. Lastly, it must be noted that the TS group essentially trained near-to-failure for the duration of the study period, which could negatively affect rapid force-generating capacity^21^. As such, other traditional-set programs that include loads and training volumes further from failure may result in different responses than the present study, and the responses may be more similar to the CS responses observed here. Nevertheless, future studies should investigate this notion, as it was not addressed in the present study. Based upon these findings, it may be warranted for powerlifters to use mixed training models which utilize both traditional and cluster sets in their resistance training programs, but it seems that traditional sets may induce more benefits for the improvements of three exercises in powerlifting including back squat, bench press, and deadlift.

## Methods

### Study design

**Figure 3:**
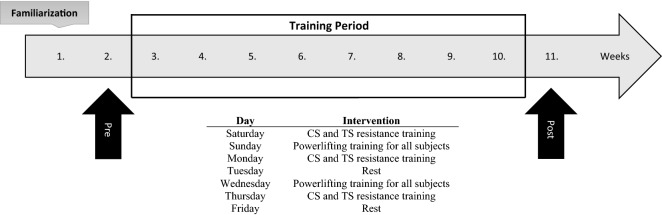
Study design. Measurements including body weight, body fat, arm and thigh circumferences, impulsive activities (i.e., vertical jump and medicine ball throw), and 1RM for the barbell bench press, back squat and deadlift exercises.

This study examined the effects of training with cluster sets or traditional sets during an 8-week training period in college-aged male powerlifters. Following a randomized-controlled, longitudinal design (Fig. 3), subjects were strength-matched across groups and performed the same off-season powerlifting program (two days per week, all using traditional sets) accompanied by a general RT program (three days per week) that differed only in the set configuration (traditional sets, or cluster sets that included extra intra-set rest periods) between July 2017 and September 2017. To continuously provide appropriate loading based on the current strength levels of the subjects, they tested every 2 weeks to modify RT intensity^22,23^. The subjects were instructed to avoid any other physical activity and to maintain their daily life habits for the whole duration of the study. Before and after the 8-week training period, the following data were collected: body weight, body fat, arm and thigh circumferences, impulsive activities (i.e., vertical jump and medicine ball throw), and 1RM for the barbell bench press, back squat and deadlift exercises.

### Participants

Twenty-four college-aged male powerlifters volunteered to participate in this study and were randomly assigned to either a group that trained using cluster sets (CS; n = 8), a group that trained using traditional sets (TS; n = 8), or a control group (CG; n = 8) (Table 3). The inclusion criteria for this study included; (1) no history of medical or orthopedic problems that compromised their participation in this study, (2) no lower extremity reconstructive surgery of any kind in the past two years or unresolved musculoskeletal disorders, (3) no use of drugs or supplements throughout 6 months before initiation of the study, and (4) experience in powerlifting for at least 3 years. All athletes were carefully informed about the experimental procedures, potential risks, and benefits associated with participating in the study and signed an informed consent document before any of the tests were performed. The Institutional Review Board at the University of Guilan approved this study (Ref. DT/12688) and it conducted in accordance with the Declaration of Helsinki II.

**Table 3:** Subject characteristics.

Baseline characteristic	CS (n = 8)	TS (n = 8)	CG (n = 8)
	Mean ± SD	Mean ± SD	Mean ± SD
Age (y)	23.6 ± 1.3	23.3 ± 0.9	23.8 ± 1.3
Height (m)	1.75 ± 0.45	1.75 ± 0.46	1.76 ± 0.31
Weight (kg)	78.6 ± 4.5	79.2 ± 4.1	77.2 ± 4.9
Training age (y)	4.7 ± 1.2	4.4 ± 1.1	4.9 ± 1.2
1RM BP (kg)	105.4 ± 11.8	95.4 ± 10.3	110.7 ± 11.2
1RM BP/BW (kg/kg)	1.34 ± 0.81	1.22 ± 0.52	1.31 ± 0.63
1RM BS (kg)	126.5 ± 9.8	126.7 ± 8.7	129.5 ± 13.7
1RM BS/BW (kg/kg)	1.56 ± 0.87	1.55 ± 0.84	1.55 ± 0.83
1RM DL (kg)	124.5 ± 9.8	121.7 ± 6.9	127.5 ± 11.7
1RM DL/BW (kg/kg)	1.51 ± 0.54	1.47 ± 0.43	1.55 ± 0.73

*CS* cluster sets, *TS* traditional sets, *CG* control group, *1RM* one repetition maximum, *BP* bench press, *BS* back squat, *DL* deadlift, *BW* body weight.

### Procedures

The powerlifters were familiarized with the strength and testing procedures during several submaximal tests one week before the measurements were taken. All tests to determine anthropometric variables, impulsive activities, and strength performance were carried out before and after the 8-week program. All tests were completed in 2 days. On day 1, each subject had their anthropometric variables (i.e. height, weight, body fat, and circumferences) assessed. After completing these measures, the subjects were assessed for their 1RM back squat and bench press. On day 2, each subject's vertical jump and medicine ball throw were measured, followed by their 1RM deadlift. During post-testing, which started five days after the final training session (i.e., the length of the pre-competition taper in this specific powerlifting club)^24^, the subjects were tested following a standardized taper (i.e., 1 day powerlifting exercises and 4 days rest) at the exact same time of day (4 to 6 P.M, post-test day) and same day of the week as the pre-test day to minimize the effect of circadian variations in the test results. To assess the reliability of the methods for determination of strength, impulsive activities, and anthropometric variables, two trials separated with 5 days were made in 12 subjects. The intraclass correlation coefficient (ICC) of these tests was r ≥ 0.95.

### Anthropometric measures

Height was measured to the nearest 0.1 cm with the use of a wall-mounted stadiometer (Seca 222, Terre Haute, IN). Body mass was measured to the nearest 0.1 kg using a medial scale (Camry, EF921, China). To determine the amount of percent body fat, skinfold thickness was measured (Saehan Caliper, model SH5020, South Korea) at 3 sites (i.e., pectoral, quadriceps, and abdominal) on the right side of the body. All the measurements were made in standing position, and percent body fat was estimated in accordance with Jackson and Pollock^25^. Circumference measures were taken for the mid-arm and mid-thigh with previously established methods^26^ whereby the right arm and thigh were measured using an anatomical tape measure to the nearest 0.1 cm during a full muscle contraction for thigh circumference and right position at side of body (i.e., relaxed in the anatomical position) for arm circumference. Each site measurement was assessed 3 times and the average of 3 trials was recorded for analysis. To determine reliability, two measurements were made in 10 subjects with 48 h apart and the ICC was 0.96 and 0.98 for the arm and thigh circumference measures, respectively.

### Strength measures

Free weight (Nebula Fitness, Inc., Versailles, OH) barbell bench press, back squat, and deadlift 1RM testing was performed according to previously described methods^2^. All subjects performed a warm-up with a light resistance for 5 to 10 repetitions. The resistance was then increased with the athlete performing 2 to 3 repetitions. From this point forward, the athlete performed 1 repetition with each progressive increased load until volitional failure was achieved^27^. The goal was to complete a maximal lift within 5 attempts. Two minutes of rest was provided between each set. Subjects were allowed one hour between different exercises. All testing was performed in the same order, which was consistent with their training history practice.

### Impulsive activities measures

Upper body impulsive activity was measured using the medicine ball throw (MBT). Prior to the MBT, the subjects performed a 10-min upper-body dynamic warm-up using active stretching and ballistic movements followed by three submaximal throws. For this test, subjects sat on the floor and a tape measure was placed on the ground at the front end of the subjects’ leg and stretched out to a distance of 10-m. The subjects were instructed to push the ball (3 kg Rubber Medicine Ball, Champion Sports, Taiwan) away from the center of their chest as far as possible, using a motion similar to a basketball chest pass. The proper angle of release to achieve maximum distance was also discussed, as instruction was shown as useful in a previous investigation^28^. A 3-min rest was given between the practices and actual throws. Subjects performed 3 trials with the 3-kg ball, with a 90-s rest between trials.

Lower body impulsive activity was measured using the countermovement jump test (CMJ) (Vertec device, Power Systems, Knoxville, Tennessee). Prior to CMJ testing, the subjects performed a standardized lower-body warm-up including three submaximal jumps. After warm-up, each subject performed three maximal CMJ, each separated by a 30-s rest period^21^. All CMJs were performed with a self-selected countermovement depth. The highest jump displacement determined from the three jumps was then used to estimate a lower body peak power (LBPP) output with the use of the equation of Harman et al.^29^ which has high intra-class correlations (ICC = 0.99) to estimating vertical jump peak powers. *Lower Body Peak Power *(*W*) = 61.9* x *(*Jump Height, cm*) + *36 x *(*body mass, kg*)* – *1822*.*

### Diet control

To avoid potential dietary confounding of results, 3-day diet recalls were completed, and the subjects were advised to maintain their customary nutritional regimen (i.e., approximately 25% protein, 25% fat and 50% carbohydrate) and to avoid taking any supplements during the study period. These nutritional aspects were monitored by a sport nutrition specialist and confirmed by personal interviews throughout the study period to monitor daily nutrient intake and hydration throughout the study. The nutrition specialist continued to meet with the subjects each week to assess adherence to their food and liquid instructions and avoidance of drugs and ergogenic supplements.

### Training program

All subjects (CS, TS, and CG) participated in the same powerlifting training for 80 to 90 min, two days per week (on Sunday and Wednesday), which included 4 to 5 sets of the bench press, back squat, and deadlift exercise with 60 to 95% of 1RM. The CS and TS groups participated in an additional three RT sessions per week on Saturday, Monday, and Thursday. Each RT session was performed between 4 and 6 P.M and lasted approximately 100 min including: 15 min warm-up, 70 min RT, and 15 min cool-down. In each RT session, the subjects performed 8 exercises including bench press, military press, arm curl, barbell arm extension, back squat, leg press, knee extension and deadlift using machine and free weights (Nebula Fitness, Inc., Versailles, OH), all of which was supervised by a researcher and strength and conditioning specialist. The RT program is presented in Table 4.

**Table 4:** Resistance training program.

			Set 1				Set 2				Set 3				Set 4		
Week	Set type	Cluster × Reps	%1RM	Cluster rest	Rest (s)	Cluster × Reps	%1RM	Cluster rest	Rest (s)	Cluster × Reps	%1RM	Cluster rest	Rest (s)	Cluster × Reps	%1RM	Cluster rest	Rest (s)
1	CS	2 × 5	70	20	120	2 × 5	75	30	120	2 × 4	80	30	120	2 × 3	85	30	180
	TS	1 × 10	70	0	120	1 × 10	75	0	120	1 × 8	80	0	120	1 × 6	85	0	180
2	CS	2 × 5	70	20	120	2 × 5	75	30	120	2 × 4	80	30	120	2 × 3	85	30	180
	TS	1 × 10	70	0	120	1 × 10	75	0	120	1 × 8	80	0	120	1 × 6	85	0	180
3	CS	2 × 5	70	20	120	2 × 5	75	30	120	2 × 4	80	30	120	2 × 3	85	30	180
	TS	1 × 10	70	0	120	1 × 10	75	0	120	1 × 8	80	0	120	1 × 6	85	0	180
4	CS	2 × 5	70	20	120	2 × 5	75	30	120	2 × 4	80	30	120	2 × 3	85	30	180
	TS	1 × 10	70	0	120	1 × 10	75	0	120	1 × 8	80	0	120	1 × 6	85	0	180
5	CS	2 × 4	80	20	120	2 × 3	85	20	120	2 × 2	90	20	120	2 × 2	95	30	180
	TS	1 × 8	80	0	120	1 × 6	85	0	120	1 × 4	90	0	120	1 × 4	95	0	180
6	CS	2 × 4	80	20	120	2 × 3	85	20	120	2 × 2	90	20	120	2 × 2	95	30	180
	TS	1 × 8	80	0	120	1 × 6	85	0	120	1 × 4	90	0	120	1 × 4	95	0	180
7	CS	2 × 4	85	20	120	2 × 3	85	20	120	2 × 2	90	20	120	2 × 2	95	30	180
	TS	1 × 8	85	0	120	1 × 6	85	0	120	1 × 4	90	0	120	1 × 4	95	0	180
8	CS	2 × 4	85	20	120	2 × 3	85	20	120	2 × 2	90	20	120	2 × 2	95	30	180
	TS	1 × 8	85	0	120	1 × 6	85	0	120	1 × 4	90	0	120	1 × 4	95	0	180

*CS* cluster sets, *TS* traditional sets, *1RM* one repetition maximum.

### Data analysis

All values are presented as mean ± standard deviation (SD). Data analyses were conducted using the Statistical Package for the Social Sciences (SPSS version 23.0 Chicago, IL). The ICC was used to determine the reliability of the measurements (2 trials performed for each measure at pre-test). Absolute and relative percentage change from pre- to post-training was calculated for all variables. A Shapiro–Wilk test was performed in order to determine if the data were normally distributed. A two-way analysis of variance with repeated measures was used to test for the effect of training (time: before, after) and training program (group: CS, TS and CG) on anthropometric measures, MBT, LBPP, and barbell bench press, back squat, and deadlift 1RM. When a significant F value was achieved, *Bonferroni *post hoc procedures were performed to identify the pairwise differences between the means whilst controlling for type I errors. The level of significance was set at *P* ≤ 0.05. Customized excel spreadsheets were used to calculate all effect size (ES) statistics. Hedge’s g (i.e., pooled SD) was utilized to calculate an effect size for all measures^30^. The magnitude of the ES statistics was considered < 0.2, trivial; 0.2–0.6, small; 0.6–1.2, moderate; 1.2–2.0, large; 2.0–4.0, very large; and > 4.0, nearly perfect^31^. The effect size is reported in conjunction with the 95% confidence interval (CI) for all analyzed measures.
